# Ranking Biomarkers of Aging by Citation Profiling and Effort Scoring

**DOI:** 10.3389/fgene.2021.686320

**Published:** 2021-05-21

**Authors:** Alexander Hartmann, Christiane Hartmann, Riccardo Secci, Andreas Hermann, Georg Fuellen, Michael Walter

**Affiliations:** ^1^Institute of Clinical Chemistry and Laboratory Medicine, Rostock University Medical Center, Rostock, Germany; ^2^Translational Neurodegeneration Section “Albrecht-Kossel”, Department of Neurology, Rostock University Medical Center, Rostock, Germany; ^3^German Center for Neurodegenerative Diseases (DZNE) Rostock/Greifswald, Rostock, Germany; ^4^Institute for Biostatistics and Informatics in Medicine and Aging Research, Rostock University Medical Center, Rostock, Germany; ^5^Institute of Laboratory Medicine, Clinical Chemistry and Pathobiochemistry, Charité –Berlin Institute of Health, Universitätsmedizin Berlin, Corporate Member of Freie Universität Berlin, Humboldt-Universität zu Berlin, Berlin, Germany

**Keywords:** aging, biomarker, health, senescence, survival

## Abstract

Aging affects most living organisms and includes the processes that reduce health and survival. The chronological and the biological age of individuals can differ remarkably, and there is a lack of reliable biomarkers to monitor the consequences of aging. In this review we give an overview of commonly mentioned and frequently used potential aging-related biomarkers. We were interested in biomarkers of aging in general and in biomarkers related to cellular senescence in particular. To answer the question whether a biological feature is relevant as a potential biomarker of aging or senescence in the scientific community we used the PICO strategy known from evidence-based medicine. We introduced two scoring systems, aimed at reflecting biomarker relevance and measurement effort, which can be used to support study designs in both clinical and research settings.

## Introduction

Aging can be described as a time-dependent multifactorial functional decline which affects the majority of living organisms (López-Otín et al., 2013). Very generally, it includes all processes that reduce health and survival of an individual (Fuellen et al., 2019). Notably, the chronological age and the “biological age” of an individual can differ remarkably (Kudryashova et al., 2020).

In this review, we give an overview of commonly mentioned and frequently used potential biomarkers of aging in clinical and research settings. A biomarker is a measurable feature (also called a marker) that predicts a biological state or condition (Siderowf et al., 2001). Biomarkers of aging are suggested to predict future health and survival better than chronological age. Further, they should be reproducible and they should also cause minimal trauma for the proband (Baker and Sprott, 1988; Fuellen et al., 2019). Until now no universally acceptable single-measurement biomarker of aging is known, and due to the complexity of the aging process, it is unlikely that a single universal biomarker of aging can be found. Many researchers believe that sets of biomarkers must be considered to predict aging-related outcomes with confidence (Earls et al., 2019; Kudryashova et al., 2020). Multiple markers may complement each other thereby improving the predictive power. In fact, recent comparisons have shown that composite biomarkers (also dubbed biomarker signatures) are potentially useful as biomarkers of aging. Belsky et al. (2018), Hastings et al. (2019) have both shown composite measures to be superior in predicting age-related outcomes. Belsky et al. (2018) tested the association of 7 different methods (3 epigenetics clocks, 3 composite biomarkers, and telomere length) with outcome measures such as physical functioning, cognitive decline, and subjective signs of aging, including aged facial appearance. The measures had a low agreement with each other. Nonetheless, one of the epigenetic clocks and all composite biomarkers were consistently, albeit modestly, related to the aging-related outcomes. In turn, Hastings et al. (2019) compared four different biomarkers of aging, three composite markers and telomere length, for their association with age-related outcomes such as physical, cognitive and perceptual functioning. Effect sizes tended to be larger for the composite biomarkers, compared to simple markers such as telomere length (Belsky et al., 2018; Hastings et al., 2019). Most bio markers including telomere length lack specificity regarding the mechanisms of aging processes (Hastings et al., 2017) and furthermore, the identification and validation of new biomarkers is important, which track aging-related changes in humans already by young adulthood and may also vary in their rate of change over time (Hastings et al., 2017).

Biomarkers of aging can be based on laboratory measurements (e.g., telomere length, epigenetic clocks) or phenotypic data (e.g., hand grip strength). Routine laboratory biomarkers are commonly measured in accredited clinical laboratories based on standardized methods, e.g., complete blood count, inflammation markers, or surrogate markers for the functional and structural status of organs such as creatinine (kidney function), bilirubin and alkaline phosphatase (liver and bile metabolism), liver transaminase (liver function and integrity), NT-proBNP (heart function), and troponin (heart structural integrity). Other molecular biomarkers are based on high-throughput analyses, which are often of unknown predictive value and are primarily used in a research context only. As molecular biomarkers we consider, in particular, all genome-level (“omics”) biomarkers. Non-molecular phenotypic biomarkers describe physiological functions of the body, specifically physical capability and organ function. Diagnostic biomarkers help to diagnose, confirm, or exclude a disease. In addition, biomarkers can be “prognostic,” for death or for the progression of disease or dysfunction, as well as “predictive,” for monitoring success or failure of some treatment. We do not explicitly distinguish biomarkers by this scheme, though we are mostly interested in prognosis.

We investigated the citation profiles of potential biomarkers of aging to gauge their “relevance.” This “relevance” is intended to be a proxy for their accuracy in predicting future health and survival. Longitudinal human studies investigating the usefulness of biomarkers of aging are lacking. Without such studies, there are no head-to-head comparative data that allow any direct ranking of markers by accuracy. We doubt that such longitudinal studies will become available soon, given the increasing number of new biomarker candidates, for which, in the short-term, validation is only possible in short or non-prospective studies, and given technical problems (lack of standardization and sampling under distinct conditions). Furthermore, each potential biomarker was assigned an effort score (e-score). The effort score should help to estimate the effort required by the measurement of a biomarker.

Many researchers have gone to great lengths to identify potential biomarkers of aging (Engelfriet et al., 2013; López-Otín et al., 2013; Xu and Sun, 2015; Wagner et al., 2016; Khan et al., 2017; Xia et al., 2017; Bai, 2018; Justice et al., 2018; Sahu et al., 2018; Dodig et al., 2019; Kudryashova et al., 2020). Most of the reviews in the field are focusing on specific subgroups of markers (e.g., senescence markers, molecular markers, omics-based markers, epigenetic markers, etc.). Our goal was to summarize the most often mentioned potential biomarkers of aging, and to suggest effort scores. The effort scores are to a certain degree subjective and dependent on circumstances such as experimental setting and location. The citation profiling scores are to some extent subjective, too, since publishing is influenced by scientific as well as other (sociopsychological, political etc.) considerations. The purpose of this review is not to propose a new comprehensive composite marker that measures reliably all aspects of aging. Nevertheless, this review may facilitate the selection of aging-related biomarkers for specific study objectives in terms of relevance and effort.

## Methods

We considered reviews, and other articles, listed at NCBI-PubMed, which contain listings of potential biomarkers. Based on these listings, we established a set of “potential biomarkers” to begin with. We were interested in biomarkers of aging in general, and in biomarkers related to (cellular) senescence. To answer the question as to whether a “potential biomarker” is indeed an aging or (cellular) senescence biomarker we used the PICO strategy known from evidence based medicine (da Costa Santos et al., 2007). The following research question was formulated:

“Is the **“*potential biomarker”*** a **biomarker** mentioned in the field of **aging** research?”

**FIGURE 1:**
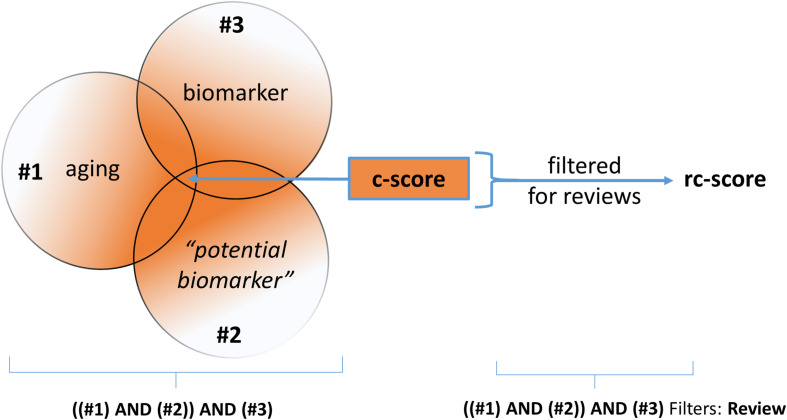
Simplified graphic of the PICO-based queries employed at NCBI-PubMed. The search strategy was conducted as “((aging) AND (*potential biomarker*)) AND (biomarker)” for establishing the “c-score” and “((aging) AND (*potential biomarker*)) AND (biomarker),” filtered for reviews, for establishing the “rc-score.”

In this context, the term “potential biomarker” is a placeholder for the reviewed biomarker. Given this question, a PICO-Scheme was built (Figure 1) and a Pubmed query was done. By the way of query processing by the Pubmed search engine, the pertinent MeSH-terms (Medical Subject Headings, NLM controlled vocabulary thesaurus used for indexing articles for PubMed) are automatically included. Due to the high number of alias names of the term “potential biomarker,” the specific MeSH-terms used by the PubMed search engine are not listed here explicitly.

The number of publications returned by the search, the count-score, is referred to as the “c-score.” As a last step the results of the PubMed search were filtered for “review;” this results in the “review count score” (rc-score). The rc-score displays how often a potential biomarker was referred to in reviews, based on the above-described NCBI-PubMed queries. For senescence markers, the first query term (#1, “aging”) was replaced by “senescence.” Potential biomarkers often mentioned in reviews are assigned a high rc-score. Potential biomarkers of aging with a high PubMed citation count are not necessarily frequently mentioned in reviews, which results in a low rc-score. The rationale behind this strategy is that reviews may fulfill at least a partial filter function for relevance. The usefulness of the rc-score is expected to be the higher, the more source articles are included. By the use of this strategy we may miss, or disvalue potential biomarkers of aging published in recent studies. Additionally, widely used and long known disease markers may be overrepresented. Further, reviews have biases such as overvaluation of some high-profile studies and undervaluation of studies published in journals with low impact. Moreover, we did not normalize for date of publication. Then again, the results of recent studies are mostly not yet further confirmed. In a final step, the potential biomarkers were sorted according to their properties in the following categories: “routine laboratory,” “research laboratory” (not epigenetic), “research laboratory” (epigenetic), “physical capability and organ function” and “senescence-related” biomarkers.

We then added an effort score for each potential biomarker. We consider the following three values:

• A low e-score (-) describes a potential biomarker which is easy to sample, to handle *and* to process (usually automated fast and reliable measurements are available from known sources). For example, blood counts from venous blood qualify, since such blood can be sampled easily, the plasma/serum can be stored at room temperature (up to 3 h for many analytes) or in a standard freezer (≥3 month for most analytes) and be processed by equipment that is regularly available in a standard diagnostic/clinical unit or research laboratory. The costs are low to moderate (≤10 €).
• A moderate e-score (–) is assigned if one step (sampling, handling, or processing) is associated with substantial extra effort for routine laboratories. This includes sampling under special conditions, a requirement for prompt sample handling, or the need for elaborate validation.
• A high e-score (—) implies elaborate sampling (e.g., biopsy, lumbar puncture, etc.), handling (e.g., storage in liquid nitrogen) and/or processing (e.g., non-routine nucleotide or protein sequencing). The financial costs are usually high.

In detail, the e-score is based on six main attributes that is, 1: easy sampling; 2: easy sample handling (storability at room temperature (up to 3 h) and/or frozen (≥3 month)); 3: automation availability; 5: routine laboratory availability; 5: costs ≤ 10 € per test; 6: degree to which the method is oblivious to confounding and interfering factors. For each attribute that is true, we incremented the score by 1, for each potential biomarker examined (see Supplementary Data 1). The following scoring system was then used:

• 6 – 5 (T): low e-score (-)
• 4 – 3 (T): moderate e-score (s.o.)
• 2 – 0 (T): high e-score (s.o.)

Just like the rc-score, the e-score is in part subjective.

## A Ranking of Biomarkers of Aging

### Routine Laboratory Biomarkers

We define a “routine laboratory” biomarker as a biomarker which is commonly analyzed in accredited laboratories based on standardized methods. It may also help to diagnose, confirm, or exclude a disease. In addition, biomarkers can be prognostic, to determine disease progression, as well as predictive, for monitoring success or failure of some treatment. In this category, cytokines such as interleukins (IL) and tumor necrosis factor alpha (TNFα) and other proteins such as C-reactive protein (CRP) belong to the most often mentioned biomarkers of aging (see Table 1). Interleukins and chemokines are secreted by leukocytes and other cell types and play a major role for the function of the immune system (Brocker et al., 2010; Ben Menachem-Zidon et al., 2011). **IL-6**, **IL-8**, **IL-15,** and **IL-1β** are often associated with aging-related inflammation, chemotaxis, and production of natural killer cells. Other inflammation-linked biomarkers of aging are high sensitive **CRP** (hs-CRP) and **TNFα** (Engelfriet et al., 2013; Wagner et al., 2016; Khan et al., 2017; Niedernhofer et al., 2017; Sebastiani et al., 2017; Bai, 2018; Justice et al., 2018; Levine et al., 2018; Tanaka et al., 2018; Dodig et al., 2019; Kudryashova et al., 2020). Elevated levels of these inflammation-related biomarkers in the blood of older individuals are risk factors for age related conditions and are often subsumed with the term “***inflammaging***” (marked in Table 1 with [IA]) (Ferrucci and Fabbri, 2018). hs-CRP is an additional less specific marker for age-dependent atherothrombosis, featuring increasing levels in advanced stages of disease. These biomarkers are influenced by inflammation-related diseases such as coronary artery disease and (type 1) diabetes mellitus and immunological diseases. The specificity of established laboratory inflammation markers is low, considering latent as well as temporarily infections as a reason for their elevation. Another frequently mentioned group of biomarkers are related to lipids, which are cardiovascular risk factors in particular, such as **total cholesterol, HDL-cholesterol, LDL-cholesterol** and **triglycerides** (Engelfriet et al., 2013; Putin et al., 2016; Wagner et al., 2016; Niedernhofer et al., 2017; Sebastiani et al., 2017; Levine et al., 2018; Tanaka et al., 2018; Dodig et al., 2019; Mamoshina et al., 2019; Kudryashova et al., 2020). Blood lipid measurement levels vary with age. Lipid levels may influence aging and are themselves influenced by aging (Walter, 2009; Johnson and Stolzing, 2019). Studies have shown that effective treatment of dysregulated lipid levels reduces mortality and morbidity. Other routine laboratory biomarkers correlate to the function and integrity of organs, which undergo age-dependent alterations. **Creatinine**, **cystatin C**, **urea,** and **albumin** are markers of renal and liver function which declines with old age (Lindeman et al., 1985; Weinstein and Anderson, 2010). Still other biomarkers discussed here are related to glucose, and are risk predictors for metabolic age-dependent conditions**: glycated hemoglobin (Hba1c)** and **glucose** (fastened or tolerance) are indicators for diabetic risk (Dubowitz et al., 2014). **Insulin**, despite high fluctuation, is an often-mentioned potential biomarker of aging. In the clinical setting, the **C-peptide** (the cleaved part of proinsulin) is most frequently measured instead of insulin due to simpler handling, less fluctuation and its equimolar amount compared to insulin. Additionally, C-peptide levels are not influenced by any insulin injections (Chandni et al., 2013). Some more routine laboratory biomarkers of aging are listed in Table 1. Taken alone, most biomarkers do not predict aging-related outcomes with high accuracy. Several studies have shown that some specific combinations of blood-based biomarkers result in more reliable predictions for “biological age” or mortality (Levine, 2013; Liu et al., 2018a). An example is the “***Phenotypic Age***,” which is based on a linear combination of chronological age and nine multi-system clinical chemistry biomarkers (marked in Table 1 with [PA]) (Liu et al., 2018a,b). With the aid of these nine blood-based biomarkers, an estimation of an individual’s “biological age” is aimed for. Further blood-based biomarkers that are part of the complete blood count are used as input for software predicting “biological age,” e.g., “**aging.AI**,” in different versions (Zhavoronkov et al., 2019).

**TABLE 1:** Frequently mentioned potential “routine laboratory” biomarkers.

Potential biomarkers	Material	Age linked processes^#^	e-score	rc-score*	c-score
Lymphocytes/WBC [CDC] [PA]	blood/EDTA	Inflammation autoimmune disorders	-	202	2240
Insulin	blood/serum	Diabetic state	–	148	1143
Glucose/glucose fastened [PA]	blood/glucose monovette	Diabetic state	-	111	1175
C-reactive protein (CRP/hsCRP) [IA] [PA]	blood/plasma	Inflammation, cancer, cardiovascular disease	-	71	1146
Cholesterol	blood/plasma	Cardiovascular disease	-	67	896
Albumin [PA]	blood/plasma	Kidney and liver dysfunction	-	65	1062
IL6 [IA]	blood/plasma	Inflammation	-	58	979
Tumor necrosis factor alpha (TNFα) [IA]	blood/serum	Inflammation, cancer	–	51	751
Hemoglobin [CDC]	blood/EDTA	Anemia, other hematopoietic disorders	-	39	471
Insulin-like growth factor 1 (IGF-1)	blood/serum	Metabolic disease	–	29	263
LDL-cholesterol	blood/plasma	Cardiovascular disease	-	24	280
Triglycerides	blood/plasma	Cardiovascular disease	-	23	498
HDL-cholesterol	blood/plasma	Cardiovascular disease	-	23	349
Creatinine [PA]	blood/plasma	Kidney dysfunction	-	19	479
Monocytes	blood/EDTA	Inflammation	-	16	378
Glycated hemoglobin (Hba1c)	blood/EDTA	Diabetic state	-	13	220
Cystatin C	blood/plasma	Kidney dysfunction	-	12	142
N-terminal prohormone of brain natriuretic peptide (NT-proBNP)	blood/EDTA	Heart failure	-	10	119
Alkaline phosphatase [PA]	blood/plasma	Liver damage, bone disorder	-	9	252
Hematocrit/RBC [CDC]	blood/EDTA	Anemia	-	8	159
D-dimer	blood/citrate monovette	Hypercoagulable state	-	8	91
IL8 [IA]	blood/plasma	Inflammation	–	7	164
Plasminogen activator inhibitor-1 (PAI1)	blood/EDTA	Prothrombotic state in cancer and other acute phases	–	6	72
Bilirubin	blood/plasma	Liver dysfunction	-	5	46
Urea	blood/plasma	Renal dysfunction	-	3	137
IL15	blood/plasma	Inflammation	–	3	55
Mean corpuscular volume/MCV [CDC] [PA]	blood/EDTA	Anemia, other hematopoietic disorders	-	2	42
Mean corpuscular hemoglobin concentration/MCHC [CDC]	blood/EDTA	Anemia, other hematopoietic disorders	-	2	32
CD4/CD8 ratio	blood/EDTA	Immune deficiency, autoimmunity	–	1	103
C-peptide (preferable to insulin)	blood/serum	Diabetic state	-	1	32
IL1-β [IA]	blood/plasma	inflammation	–	1	5

* rows are sorted by rc-score.			[IA] = inflammaging		
*^#^ frequently mentioned general or disease-linked processes.*			[PA] = Phenotypic Age		
			[CDC] = complete blood count		

### Non-epigenetic Research Laboratory Biomarkers

A research laboratory biomarker is a laboratory biomarker lacking the routine validation and/or standardization of a clinical laboratory biomarker. These markers often use nucleic acids (DNA and RNA). A frequently discussed biomarker of aging is the **telomere length** (López-Otín et al., 2013; Wagner et al., 2016; Khan et al., 2017; Niedernhofer et al., 2017; Xia et al., 2017; Bai, 2018; Dodig et al., 2019; Vaiserman and Krasnienkov, 2020). Telomeres are repeats of a hexametric DNA sequence capping the end of chromosomes preventing DNA damage (Aubert and Lansdorp, 2008). Telomeres shorten with each cell division or due to cell stress. This attrition ultimately leads to cellular senescence and can thus function as a biomarker for replicative aging in mitotic cells. Specifically, leukocyte telomere length is used as a potential biomarker for healthy aging (Bekaert et al., 2005; Lulkiewicz et al., 2020), whereby shortened telomeres may represent cellular exhaustion and/or increased cell stress for leukocytes or other cells in the body as a surrogate; however the usefulness of telomere length as a biomarker of aging is discussed critically. Kahl & Allen et al. summed up and described different methods measuring telomere length, covering imaging based methods (TCA, TRF, Q-FISH, Flow-FISH) and PCR-based methods (qPCR) (Kahl et al., 2020). Another very good overview is provided in the review of Lai et al. (2018), comparing known methods and additionally describing methods to measure the shortest telomeres (STELA, TeSLA). Telomere length measurements are characterized by poor standardization and limited comparability. Moreover, telomere length in leucocytes is only partially a surrogate marker for telomere length in other organs. Telomere length is classified as highly relevant according to the rc-score, which exemplifies the limitations of such a score. The telomere length is certainly very interesting from a physiological point of view, but whether it can serve as a relevant (or even accurate) biomarker is questionable. Telomere shortening can only be counteracted in a few cells (germ cells, stem cells, some immune cells) by **telomerase**, an enzyme that is able to lengthen the telomeres (Shay, 2016). Telomerase is induced in most tumor cells, which renders its induction risky. Nevertheless, telomerase activity plays an important role in longevity. A study showed that centenarians have a particularly active telomerase in T-cells compared to healthy 67 – 80 year old donors (Tedone et al., 2019), which raises the question if telomerase activity could be a biomarker for aging. Another DNA-linked potential biomarker of aging is the degree of **DNA damage** (Wagner et al., 2016; Khan et al., 2017; Niedernhofer et al., 2017; Dodig et al., 2019). DNA damage accumulates with age, fostering the development of age-related pathologies such as malignancies, cellular senescence and inflammation (Da Silva and Schumacher, 2019). In particular, mitochondrial DNA damage is a factor leading to **mitochondrial dysfunction**, which is also often related to aging processes (López-Otín et al., 2013; Khan et al., 2017; Niedernhofer et al., 2017; Sahu et al., 2018; Dodig et al., 2019). Mitochondrial DNA has less repair capacity and a higher mutation rate compared to nuclear DNA (Haas, 2019). Mitochondrial, metabolic and respiratory dysfunction can, in addition to exogenous stress, lead to the production of excess **reactive oxygen species (ROS)** (López-Otín et al., 2013; Wagner et al., 2016; Santos et al., 2018; Dodig et al., 2019; Kudryashova et al., 2020). Potential negative effects of excess ROS are dysregulated protein homeostasis, accumulation of oxidative modified proteins and advanced glycation/lipid peroxidation end products and loss of function of cellular protein maintenance systems. It has been shown that **autophagy** is another modulator of aging processes. A tissue-specific overexpression of autophagy genes can be sufficient to extend lifespan by preventing the accumulation of dysfunctional cellular components (Simonsen et al., 2008; Hansen et al., 2018; Kumsta et al., 2019). Such hallmarks of aging as discussed here were observed in various organs and tissues (Stadtman and Berlett, 1997; Baraibar and Friguet, 2013; Davies et al., 2017). In response to mitochondrial dysfunction, **growth differentiation factor 15 (GDF15)** may be generated, protecting tissues against inflammation by suppressing T-cell activation and mediating release of cytokines (Moon et al., 2020). On this basis, GDF15 was suggested as a potential aging biomarker (Justice et al., 2018; Tanaka et al., 2018; Basisty et al., 2020; Sebastiani et al., 2021). **TGF-β** and **GDF11** (from the same protein superfamily) are also regarded as proteins playing a role in aging-associated cellular senescence, frailty, stem cell aging and fibrosis as well as surgical risk in older adults (Schafer et al., 2016; Khan et al., 2017; Niedernhofer et al., 2017; Bai, 2018; Dodig et al., 2019; Tominaga and Suzuki, 2019). Circulating biomarkers based on **extracellular vesicles (EVs)**, including exosomes, microvesicles and apoptotic bodies, are moving into focus for the prediction of age-related diseases (Yáñez-Mó et al., 2015; Kalluri and LeBleu, 2020; Noren Hooten, 2020). Moreover, changes in the community composition of the **skin microbiome** have been related to age (Kim et al., 2019), and more precise information is obtained when considering the **gut microbiome** (Maynard and Weinkove, 2018; Guest, 2019; Askarova et al., 2020). In general, there is now ample evidence that microbiome dysbiosis is associated to aging and longevity (Kim and Benayoun, 2020). Other frequently mentioned potential research laboratory biomarkers of aging are listed in Table 2.

**TABLE 2:** Frequently mentioned potential “research lab” biomarkers based on non-epigenetic measurements.

Potential biomarkers	Material	Methods	Age linked processes^#^	e-score	rc-score*	c-score
Telomere length (TL):			Morbidity, mortality, cell stress		191	932
Average TL	DNA	Q-PCR, TRF, TCA		–		**
TL structure	DNA	Q-FISH, Flow-FISH		—		**
Shortest TL	DNA	STELA, TeSLA		—		**
DNA damage	DNA	Various methods	Morbidity, mortality	–	174	713
Reactive oxygen species (ROS)	Tissue mitochondria	Various methods	Morbidity, cell stress, DNA/protein damage	—	168	712
Mitochondrial dysfunction	living cells, mitochondrial DNA	Various methods	Morbidity, mortality, neurodegenerative diseases	—	86	289
EVs (extracellular vesicles)	blood/plasma, liquor, cell culture supernatant	Immuno-histochemistry Western Blot, FACS	Cellular senescence, cancer	—	65	194
Autophagy	cells, cell extract	Electron microscopy immunoblotting flow cytometry	Morbidity, cancer, Parkinson’s and Alzheimer’s disease	—	46	207
Transforming growth factor beta (TGF-β)	blood/serum	ELISA	Inflammation, fibrosis, cellular senescence, cancer	–	45	315
Telomerase activity	cell extract, DNA	PCR-ELIDA, TRAP	Morbidity, mortality, tumor progression	—	41	169
Gut microbiome	fecal specimen	Next generation sequencing	Morbidity, mortality	–	29	101
α-Klotho	blood/plasma tissue	Immuno-histochemistry ELISA	Morbidity, mortality, renal function	–	20	107
Adiponectin	blood/plasma blood/EDTA	ELISA	Morbidity, mortality, frailty, metabolic syndrome, liver cirrhosis, diabetes type 2	-	14	217
Sirtuin 1 (SIRT1)	blood/serum	ELISA immuno-histochemistry PCR	Morbidity, mortality, inflammation, cancer	–	12	112
Growth differentiation factor 15 (GDF15)	blood/plasma	Proteomics immunoassays	Morbidity, organ damage (liver, heart, kidney)	–	12	63
Sirtuin 6 (SIRT6)	blood/serum	ELISA immuno-histochemistry PCR	Morbidity, mortality, diabetic risk, arthritis	–	4	50
Growth differentiation factor 11 (GDF11)	blood/plasma	Proteomics immunoassays	Morbidity	–	3	22
CXCL1	blood/plasma	Immunoassays, ELISA	Immune response, inflammation, cancer, Alzheimer’s disease	–	0	15
Skin microbiome	skin swab	Next generation sequencing	Morbidity, mortality	–	0	4

* rows are sorted by rc-score. ** included in the c-score of TL. ^#^ frequently mentioned general or disease-linked processes.

### Epigenetic Research Laboratory Biomarkers

The epigenome is a dynamic system playing a major role in aging. Methylation of the DNA (DNAm) and histone modifications ensure appropriate high fidelity gene expression; both change with chronological age and with chronic diseases over time. Even if it is currently not known to what extend these changes cause aging, they can be useful, e.g., for chronological age prediction (Ashapkin et al., 2019). Generally, aging is associated with global hypomethylation and local hypermethylation. For the analysis of DNA methylation, various so-called epigenetic clocks were developed. Famous examples for first generation epigenetic clocks are the **Horvath clock**, **Weidner Clock,** and **Hannum clock** (see Levine, 2019). Basically, these clocks are considering specific sets of CpG sites with respect to their DNA methylation status, as a molecular correlate to predict chronological age (Horvath and Raj, 2018; Bell et al., 2019). Hannum’s clock is based on blood samples and uses 71 CpG sites measured from the Illumina 450k array. Age-related shifts in blood cells are *per se* informative for changes in chronological age (Hannum et al., 2013; Bell et al., 2019), but they are also considered by some epigenetic clocks. The Horvath clock is specifically designed to be used across multiple tissues, whereby it captures 353 CpG sites on a Illumina 27k array (Horvath, 2013; Bell et al., 2019). Second-generation epigenetic clocks learn to associate clinical data with methylation status, e.g., the **DNAm PhenoAge** (Levine et al., 2018) or **DNAm GrimAge** (Lu et al., 2019). More specifically, second-generation epigenetic clocks such as GrimAge and PhenoAge were developed to learn biological endpoints (which in turn are suggested to relate to “biological age”) directly. Recently, it was shown that cytosines, whose methylation levels change with age across mammalian species, are involved in mammalian developmental processes, suggesting that aging is indeed evolutionarily conserved and coupled to developmental processes (Lu et al., 2021). Histone modifications such as **H4K16 acetylation**, **H4K20 methylation**, **H3K4 methylation**, **H3K9 methylation** and **H3K27 methylation** were also proposed as epigenetic chronological age predictors (López-Otín et al., 2013; Moskalev, 2019), and these modifications can be influenced by ROS (Wu and Ni, 2015). However, data regarding specific outcomes are scarce. Histone modifications and DNA methylation are closely related to **chromatin remodeling** and changes in chromatin architecture (Oberdoerffer and Sinclair, 2007; López-Otín et al., 2013). The above mentioned EVs also carry **extracellular RNA (exRNA)** which changes with age (Dluzen et al., 2017; Noren Hooten, 2020). Other types of RNA such as **microRNAs (miRNA)**, that function in RNA silencing and posttranscriptional regulation of gene expression, and which are often isolated from peripheral blood mononuclear cells (PBMCs), are also reflecting aging and are used to predict age-related diseases (Evans et al., 2010; Noren Hooten et al., 2010; Reid et al., 2011; Kumar et al., 2017). Examples for age-related miRNAs are: miR-34a, miR-9, miR-132, miR-212, miR-21, miR-96, miR-145 (Halper et al., 2015; Budzinska et al., 2016; Owczarz et al., 2017; Hadar et al., 2018). Frequently mentioned epigenetic based biomarkers are covered in Table 3.

**TABLE 3:** Frequently mentioned potential “research lab” biomarkers based on epigenetic measurements.

Potential biomarkers	Material	Methods	Prediction	e-score	rc-score	c-score*
DNA methylation and aging clocks:					n.a.	2158
Horvath’s clock	DNA (broad spectrum of tissues)	DNA methylation analysis	Chronological age	–	n.a.	214
Hannum’s clock	DNA (blood)		Chronological age	–	n.a.	190
DNAm GrimAge	DNA (blood)		Biological age	–	n.a.	31
DNAm PhenoAge	DNA (blood)		Biological age	–	n.a.	26
Weidner clock	DNA (blood)		Chronological age	–	n.a.	8
EpiTOC	DNA (blood)		Biological age	—	n.a.	2
miRNA (microRNA)	RNA (blood/plasma PBMCs)	Next generation sequencing microarrays	Morbidity, mortality	—	198	635
Non-coding RNA expression profiles	RNA	RNA sequencing	Chronological age	—	167	602
exRNA (extracellular RNA)	blood/plasma	Next generation sequencing	Morbidity, mortality	—	25	119
Histone modifications:					36	73
H4K20 methylation		DNA methylation analysis mass spectrometry, HPLC, ChIP Immunohisto-chemistry	Cell stress	—	n.a.	n.a.
H4K16 acetylation				—	n.a.	n.a.
H3K4 methylation	protein extract			—	n.a.	n.a.
H3K9 methylation	from tissue DNA			—	n.a.	n.a.
H3K27 methylation				—	n.a.	n.a.
Chromatin remodeling	DNA	Chromatin remodeling assays	Chronological age	—	13	26

* rows are sorted by c-score. n.a.: not assigned due to high variation of terminology in literature.

### Other Aging Biomarkers: Physical Capability, and Organ Function

Physical and cognitive function are important markers for aging processes (Fuellen et al., 2019), as are anthropometric measurements. For example, brain function and integrity are influenced by aging and aging-associated diseases. Aging encompasses changes at the structural, functional, and molecular levels of most cells, tissues and organ systems. Gradual loss of the maintenance functions of tissues is a characteristic of aging (López-Otín et al., 2013). Non-blood aging markers are not the primary focus of this review. However, due to the ease of implementation, certain analyses can supplement studies in which general metabolic and physiological age aspects need to be measured. These tests may include **grip strength** or easy to perform locomotor function tests as **walking speed**, **timed up and go test** or the **standing balance test**. As aging is associated with body composition, biomarkers such as **BMI** or fat and muscle indices should be recorded. **Bone mass** declines with age in both men and women and may be analyzed (Khosla et al., 1996; Khosla et al., 2006; Riggs et al., 2008; Keaveny et al., 2010). Frequently used other anthropometric markers are **muscle mass**, **waist circumference** (Wagner et al., 2016), and **(systolic) blood pressure** (Pinto, 2007; Crimmins et al., 2008). These measurements are mostly carried out without much effort. Most of these markers are also closely related, and they predict frailty in particular. Frailty is age-dependent and often associated with chronic disorders, resulting in an increasing need for diagnostic, nursing, and therapeutic interventions. Grip strength is a predictor of frailty, all-cause mortality and morbidity (Syddall et al., 2003). Strength itself may provide protection against mortality (Rantanen et al., 2000). With age, a decline in physical and cognitive function is frequently observed, as can be seen in the lifespan data of athletes in comparison to controls (Donato et al., 2003; Baker and Tang, 2010; Harridge and Lazarus, 2017). This is observed for muscle mass but also for physiological changes in organ systems leading to age-related diseases (Boss and Seegmiller, 1981). Additionally, various non-invasive methods were proposed to monitor the cardiovascular system and the vascular wall structure and elasticity, including electrocardiogram (ECG), intima media thickness ultrasonography and ultrasound techniques to evaluate endothelium-dependent vasodilation (EDV). Many if not all aspects of cognitive function change with age, which can be measured in complex tests or in rather simple questionnaires. Frequently mentioned non-blood and biomarkers are covered in Table 4.

**TABLE 4:** Frequently mentioned potential non-blood physical capability and organ function biomarker.

Potential biomarkers	Method	Age linked processes^#^	Domain	e-score	rc-score*	c-score
**Physical capability**						
Grip strength	Physical exam	Mortality, morbidity	Strength	–	11	229
Walking speed	Physical exam	Mortality, morbidity	Locomotor function	–	3	106
Standing balance	Physical exam	Mortality, morbidity	Balance	–	1	26
Timed up and go test	Physical exam	Mortality, morbidity	Locomotor function	–	0	11
**Organ function**						
Atherosclerotic lesions	IMT, ultrasound	Mortality, CAD	Cardiovascular system	–	158	680
Muscle mass	MRI	Mortality, cardiovascular risk	Body composition	–	81	495
Systolic blood pressure	Auscultatory method	Mortality, cardiovascular risk	Cardiovascular system	–	65	844
Cognitive function	Various	Mortality, morbidity	Brain function	—	56	581
Body mass index	Calculated	Mortality CAD	Body composition	–	24	1280
Bone density	Bone density test	Mortality, morbidity	Body composition	–	17	84
Lung function	Spirometry	Mortality, morbidity	Respiratory system	–	16	84
Waist circumference	Tape measure	Mortality, cardiovascular risk	Body composition	–	3	202
**General well being**						
Health assessments	Questionnaire	Mortality, morbidity	General	–	n.a.	n.a.

* rows are sorted by c-score. ^#^ frequently mentioned general or disease-linked. n.a.: not assigned due to high variation of terminology in literature.

### Senescence-Related-Biomarkers

Cellular senescence is a cell state characterized by the cessation of cell division, reached through a combination of telomere shortening, oxidative stress and oncogenic stress. It can also be induced by each of these factors alone, and by DNA damage signaling pathways, with ATM and ATR as primary sensors of DNA double and single-strand damage. As a species-specific aging mechanism, telomere attrition limits the number of divisions. The successive shortening of the chromosomal telomeres with each cell cycle (caused by the so-called end replication problem and often referred to as replicative senescence) is observed in large long-lived species and cooperates with other aging mechanisms to activate the senescence program. These signaling pathways are funneled down to activate the p53 protein, the Rb protein, or both. Once the senescence program is activated, a series of changes in morphology, function, and gene expression takes place, associated with autocrine and paracrine effects of secreted cytokines, macromolecular damage, and altered metabolism (Gorgoulis et al., 2019). The limitation of investigating “senescence-related” biomarkers lies in the cumbersome extraction of appropriate patient samples. Furthermore until now, there is no clinically validated senescence-related biomarker available. Indirect biomarkers of cellular senescence can be measured in blood samples, such as markers of proliferation status or components of the SASP (Castleab et al., 1999). However, these measurements are non-specific and only a proxy of the senescence status of the cells providing the sample. Moreover, they cannot reflect the senescence status of the entire organism (Schafer et al., 2020; Yousefzadeh et al., 2020). Additionally, often it is not clear from which tissue(s) the markers originate, since markers found in blood could originate from almost anywhere. The proliferation status of senescent cells can also be gauged by the expression of various cell-cycle-related markers (p16, p21, p53) (Jung et al., 2010; Baker et al., 2011; Burd et al., 2013; Qian and Chen, 2013). On one hand, cellular senescence is a cause or bystander of many age-related diseases contributing to inflammation and/or tumorigenesis (Milanovic et al., 2018; Song et al., 2020). On the other hand, cellular senescence and its generally irreversible loss of proliferative potential is considered necessary for tissue remodeling during development, tissue homeostasis, wound healing as well as for tumor prevention (Ovadya and Krizhanovsky, 2018). Identification and characterization of cellular senescence markers receives more and more attention. However, the difficulty in characterizing cellular senescence by biomarkers and the lack of blood markers or other easily accessible specimen still limit the usefulness of the concept (Khan et al., 2017; Hernandez-Segura et al., 2018).

**FIGURE 2:**
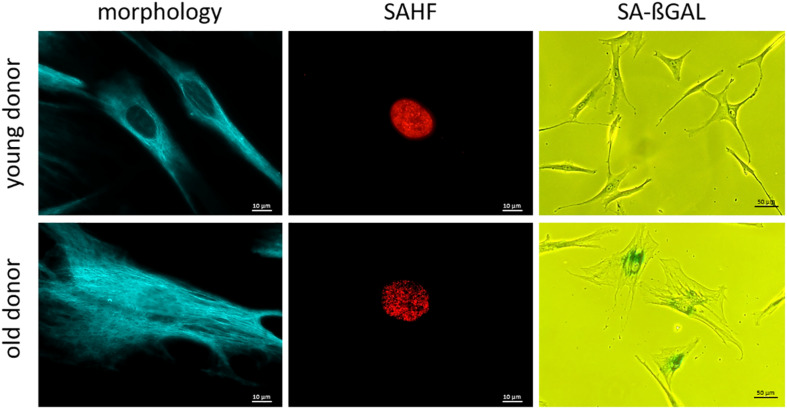
Representative microscopy pictures of cellular senescence biomarkers. Note the clear difference in overt morphology due to age of the respective individual at biopsy (cells are larger in size and more flattened; cells were immunostained for vimentin.) **SAHF** are formed in old fibroblasts and are enriched in heterochromatin markers. (Cells were immunostained for H3K9Me3.) **SA-ßGal** activity increases with age at pH 6.0. (Cells were treated with X-Gal to make SA-βGal visible in senescent cells.); scale bar: 10 μM for morphology and SAHF column, 50 μM for SA-βGAL column.

The senescence-associated secretory phenotype (SASP) is characterized by an enrichment of various inflammatory markers (Ghosh and Capell, 2016; Hernandez-Segura et al., 2018; Basisty et al., 2020) and can be detected in serum or EDTA plasma of probands using ELISA (Tanaka et al., 2018), which is readily available. Some important SASP markers are: interleukins (IL-6, IL-7, IL-8, and IL-15); chemokines (CCL3, CCL4) as well as growth factors (GDF-15 and activin A) (Schafer et al., 2020). Nevertheless, the secretion of these markers is highly heterogeneous and regulated at many levels, making it difficult to consider them as well-standardized biomarkers of cellular senescence (Hernandez-Segura et al., 2017). Assignment of inflammatory markers to the SASP or to another aging-related physical or physiological status (e.g., inflammaging) as well as to other diseases with permanent inflammation status (e.g., infections, tumors) is often difficult (Serrano et al., 1997; Barbé-Tuana et al., 2020). For the investigation of some cellular senescence markers, it is advantageous to have cells in culture, e.g., fibroblasts or PBMCs (Migliaccio and Palis, 2011; Riedhammer et al., 2016; Wang and Dreesen, 2018). For fibroblast isolation, a punch skin biopsy (Zuber, 2002; Vangipuram et al., 2013) may be taken, which may represent a notable trauma for the probands (Vangipuram et al., 2013). Another disadvantage of fibroblast cell culture is the long period of culturing of around 50 days, before investigations can be completed (Vangipuram et al., 2013), the difficult standardization, and other confounders such a population doublings in cell culture conditions. Consequently, this procedure is only used for research purposes. Much more easily done is the isolation and cultivation of PBMCs from blood samples including lymphocytes (T-cells, B-cells, NK-cells) (Migliaccio and Palis, 2011; Riedhammer et al., 2016). Senescent cells, especially fibroblasts in culture, become larger in size, flattened in shape (Figure 2) and can display a disorganized nuclear envelope mediated by reduction of lamin B1 expression (Nishio et al., 2001; Freund et al., 2012). These features of cellular morphology are features of senescent cells in general. Moreover, progerin (a mutated form of lamin A) associated with the premature aging syndrome Hutchinson-Gilford (DeBusk, 1972) can be detected in fibroblasts, also at low levels due to non-premature aging (Scaffidi and Misteli, 2006). A recent study has shown that elevated blood levels of progerin can be detected in people with obesity, suggesting a cause for premature aging of the cardiovascular system. Therefore, progerin might be measured in blood samples and be adapted for diagnostic measurements (Messner et al., 2019). Another marker of cellular senescence, which can be investigated in cultured cells (e.g., based on punch skin biopsy), are senescent-associated histone foci (SAHF). These darker regions within the nucleus of senescent cells can be detected as compacted DNA foci. While DNA staining of healthy and young human cells is relatively uniform, senescent cells show up to 50 punctuated DAPI-stained DNA foci (Narita et al., 2003; Aird and Zhang, 2013). Additionally, SAHF are enriched in markers of heterochromatin (H3K9Me3 and HP1γ) (Figure 2; Sharpless and Sherr, 2015). Furthermore, senescent cells usually have an increased lysosomal content, which can be detected cytochemically by measuring senescence-associated β-galactosidase (SA-βGal) activity at a pH of 6.0 (Debacq-Chainiaux et al., 2009; Hernandez-Segura et al., 2018; Figure 2). Frequently mentioned senescence related biomarkers are covered in Table 5.

**TABLE 5:** Frequently mentioned biomarkers (routine or research laboratory) associated with cellular senescence.

Potential biomarker		Material and Methods	e-score	rc-score*	c-score
SASP				442	2646
	Cytokines (e.g., IL-6, IL-7, IL-15)	ELISA from Serum or EDTA plasma samples proteomics	–° –° –° –	n.a.	n.a.
	Chemokines (e.g., IL-8, CCL3, CCL4)			n.a.	n.a.
	Growth factors (e.g., GDF-15, activin A)			n.a.	n.a.
Cell cycle arrest	p53	qPCR from blood samples/staining of cultured cells/flow cytometry NGS/microarray	– – –	66	561
	p16			27	422
	p21			21	435
SA-βGal		Microscopy/flow cytometry	—	9	359
SAHF	Histone fragments (H3K9Me2, HP1γ)	DAPI/heterochromatin staining	—°	3	19
Lamin B1		Immunohistochemistr Western Blot	—	0	12
Cell morphology (e.g., progerin)	Cell shape	Microscopy of cultured cells	—	n.a.	n.a.

* rows are sorted by rc-score. ° on average (detailed in Supplementary Table 1, 5). n.a.: not assigned due to high variation of terminology in literature.

In summary, there is currently no biomarker for cellular senescence that can easily be used for diagnostics or prediction. Therefore, in the future, more attention should be paid to the research and establishment of diagnostically applicable biomarkers of cellular senescence. Potentially, measuring senescence markers after cell isolation by flow cytometry can be further developed for use in diagnostics (Adewoye et al., 2020).

### Transcriptomic Biomarkers

Transcriptomic (gene expression) changes also accompany the aging process, and transcriptomic clocks were trained to predict chronological age. Some clocks were trained on samples belonging to a specific tissue, e.g., the clocks by Peters et al. and Huan et al. are based on whole blood or PBMC, the clocks by Fleischer et al. and LaRocca et al. are based on skin fibroblasts, while the one by Mamoshina et al. (2019) is based on muscle tissue, and the clocks by Ren and Kuan (2020); Shokhirev and Johnson (2020) are based on multiple tissues (Peters et al., 2015; Fleischer et al., 2018; Huan et al., 2018; Mamoshina et al., 2018; LaRocca et al., 2020; Ren and Kuan, 2020; Shokhirev and Johnson, 2020). Most of these clocks are taking mRNA as input, while some employ microRNAs (Huan’s clock) or repetitive elements (LaRocca’s clock). Shokhirev and Johnson’s pan-tissue transcriptomic clock is based on a large-scale meta-analysis of transcriptomic data from the Sequence Read Archive. 3060 multi-tissue samples were used as input, and a random forest model was able to learn chronological age with high accuracy (Shokhirev and Johnson, 2020). On a practical level, the e-score of transcriptomic clocks strongly depends on the tissue used for the analysis. Perhaps the best specimen in this context is whole blood or its components. Peripheral blood mononuclear cells (PBMC) are easily accessible as well, and PBMC transcriptomes were measured in the context of nutrigenomic interventions and proved to be sensitive to these (Afman et al., 2014; Herrera-Marcos et al., 2017; Bottero and Potashkin, 2020).

### Preanalytics and Methodology Reporting

Appropriate preanalytics and exact description of sampling and methodology are critical for reliable results and reproducibility, particularly the use of validated tests and procedures. Details are beyond the scope of this review and are summarized elsewhere (Rai and Vitzthum, 2006). These include blood draw, blood collection, blood processing and storage. We recommend, whenever possible, a morning (before 10 am, and fasting) blood draw to control diurnal fluctuation; Mondays should be avoided due to weekend-specific confounders. For blood collection a 21-gauge needle and slow aspiration is preferred to avoid the activation of coagulation. Needle material and tube material may impact assays, e.g., in the measurement of trace elements. A standardized description of venipuncture and sampling is strongly recommended. There are advantages and disadvantages for serum and plasma respectively (Rai and Vitzthum, 2006). If possible, both serum, heparin plasma, double centrifuged citrate plasma and, if necessary, EDTA blood (e.g., for DNA preparation, telomere length measurements, etc.) should be collected and prepared in fractions, and, if necessary/possible, frozen. Blood counts and other highly standardized routine methods should be performed immediately. In general, tests with high inter-assay variability should be processed batch-wise based on initially frozen samples, provided the samples can be frozen for the respective test. Data on the stability of the frozen samples and on potential confounding by the freeze thawing procedures should be recorded. The position of the probands during blood drawing (standing, lying, sitting) influences almost all analyte values (due to water shifts between vessel and interstitial space). Sampling from probands at a stable position (horizontal or sitting for some minutes) is recommended. Serum/plasma should not be in contact with blood cells for more than 2 h (Kiechle, 2010). Long-term storage should be at –80°C or in liquid nitrogen (Rai et al., 2005).

## Conclusion

Aging is a complex process and not fully understood. In this review we propose the “rc-score” and the “e-score” as tools for gauging the suitability of biomarkers of aging, with a focus on clinical settings. Together the two scores reflect the presumed relevance of a potential biomarker of aging, and the effort needed for its measurement. The “e-score” must be seen in relation to the equipment and possibilities of the laboratory performing the measurements. Routine blood biomarkers and easy-to-measure phenotypic markers such as blood pressure often correlate well with age-dependent organ/metabolic dysfunction, including cardiovascular, renal, or diabetic risk. The most cited non-epigenetic biomarkers are telomere length, amount of DNA-damage and mitochondrial dysfunction, reflecting aging-related changes on the genome and cellular level. Telomere length is classified as the most relevant according to rc-score, which exemplary shows the limitations of such a score. Telomere attrition is very interesting from a physiological point of view, but whether it can serve as an accurate biomarker is still questionable due to methodological problems. Novel telomere analyses such as TESLA have yet to be validated. Molecular markers such as cytokines/chemokines and sirtuins show a relatively low rc-score but are of clinical and scientific interest. The strong presence of BMI in reviews might also be attributed to the fact that potentially better markers such as the formal criteria of metabolic syndrome were not considered in many studies because the effort is higher for these. Methylation of CpG sites is among the most interesting candidate biomarkers of aging, but it awaits further validation in longitudinal studies. Functional decline affects all types of tissues and has a negative effect on grip strength and mobility, which can both be used as biomarkers of aging. Cellular senescence is a fundamental part of the aging process. However, it is difficult to analyze to date in a clinical setting due to difficulties in sampling and specificity. A combination of routine laboratory, epigenetic, non-epigenetic and physical capability and organ function biomarkers, and possibly senescence markers, may be the key to a valid composite biomarker of aging. Yet, a standardized (composite) biomarker of aging that specifically measures all important aspects of the aging process has not yet been found.

### Clinical Get Home Message

The general question of which biomarkers should be used in clinical trials to study aging and (cellular) senescence remains difficult and clearly requires further systematic longitudinal studies. Nevertheless, there are numerous potential markers, which differ concerning their difficulty to sample, to handle and to process, including significant differences in costs. Several parameters can be used to select biomarkers of aging for clinical trials, and we point out some specific issues in Table 6.

**TABLE 6:** criteria to select biomarkers of aging in clinical trials.

Cohort Size:	Large cohorts require biomarkers that are easy to extract and process at low cost (e.g., serum markers). Studies with smaller cohorts and more specific aging-associated questions may require (and can afford) difficult-to-use and/or more expensive markers (e.g., fibroblast cultures, measurement of telomere length or the methylation level of CpG islands).
Cohort type:	Depending of the aims of the trial, usually reflected by inclusion criteria, it is often not appropriate to consider biomarkers which are usually used as markers for specific tissue damage or organ failure (e.g., creatinine, cystatin C, Pro-BNP) or markers that reflect a general activation of immunological processes such as CRP and IL6 or markers that reflect a higher risk for typical age-related diseases such as lipids, HbA1c or other cardiovascular risk factors. Additionally, organ specific markers should be controlled because these can be strong confounders in a study. A possible strategy to increase the informative value for all aging aspects could be the combination of organ-specific and more general markers.
Compartment of disease:	If the disease (or dysfunction) that is specifically considered in a trial features strong effects not in general but in distinct compartments (organs, tissues, combinations of these, or parts thereof), e.g., the brain, the overall question is which compartment to sample for biomarker analysis, e.g., peripheral blood vs. cerebrospinal fluid. Markers in the blood can often but not necessarily be attributed to more general aging processes.
Assessment of potential pitfalls:	Even if easy-to-handle biomarkers have a high sensitivity for aging-related processes, they often lack clinical specificity. This is true for many inflammatory markers (e.g., CRP, interleukins), which are more valuable markers of aging in populations without an overrepresentation of infections. For most questions acute infection must be ruled out by standard criteria (fever, feeling unwell, B-symptoms, etc.). Specific tissue/organ checks (e.g., physical examination, echocardiography etc.) can be added to rule out acute diseases. Strictly speaking, the biomarkers excluded on this basis may also reflect some acceleration of aging-related processes. However, they are less relevant than biomarkers reflecting more general aspects of aging, and, more importantly, they would lead to misinterpretations in individual patients. Furthermore, in addition to standard preanalytics precautions such as control of patient’s position, application of the tourniquet, fasting vs. non-fasting and diurnal fluctuations, special aspects must be taken into account. For example, measurements that may be influenced by habits such as exercise should not be done on Mondays; exercise on weekends may influence cytokine levels, etc. In general, the same days should be used for all participants and all longitudinal time points.
Future directions:	There is a strong need to investigate biomarkers of aging more systematically. This should include promising markers such as the methylation of CpG islands and the standardization for specific sampling procedures (e.g., of peripheral blood cells for specific measurements) and the clarification as to whether and in what context acute disease markers, which at the same time can also reflect chronic processes of aging, are useful biomarkers of aging. Furthermore, biomarkers might be put together into composite markers, also known as “aging panels.” Finally, the assessment of very sophisticated but highly informative measures with high potential validity to monitor aging such as MRI (“Brain age“) or PET-Scans (e.g., TAU-PET, detecting the continuous increase of TAU deposition in temporo-parietal-occipital lobes) should be considered (Sowell et al., 2003; Cole et al., 2015; Schöll et al., 2016).

## Author Contributions

AHa, CH, and GF wrote the manuscript with input of RS, AHe, and MW. AHa did the analyses underlying the preparation of the tables. All authors approved the final version of the manuscript and made substantial, direct and intellectual contribution to the work, and approved it for publication.

## Conflict of Interest

The authors declare that the research was conducted in the absence of any commercial or financial relationships that could be construed as a potential conflict of interest.
