# Supplementary material for: Ranking Biomarkers of Aging by Citation Profiling and Effort Scoring
Source: Front Genet. 2021 May 21;12:686320. doi: 10.3389/fgene.2021.686320 (PMC8176216; doi:10.3389/fgene.2021.686320)
Supplement: Supplementary file 1 [file Data_Sheet_1.PDF]

Supplementary Material

1 Supplementary Data

S1.) The e-score scoring system

As attributes for each potential biomarker, we considered the following:

1. sampling (standard venous blood collection reflects low effort)
2. sample handling (storability at room temperature (up to 3 h) and/or frozen (≥ 3 month))
3. automation availability
4. routine laboratory availability
5. costs ≤ 10 € per test, yes or no
6. degree to which the method is oblivious to confounding interfering factors, high or low

For each attribute it was checked if it is true (T) or false (F) for the examined potential biomarker. The following scoring system was used:

- 6 – 5 (T): low e-score (-)
- 4 – 3 (T): moderate e-score (--)
- 2 – 0 (T): high e-score (---)

table S1.1: Scoring system for e-score assignment ("routine laboratory" biomarkers)

biomarker	1	2	3	4	5	6	e-score
lymphocytes / WBC	T	F	T	T	T	T	-
glucose	F	T	T	T	T	T	-
CRP	T	T	T	T	T	T	-
insulin	F	F	T	T	T	T	--
albumin	T	T	T	T	T	T	-
IL6	T	T	T	T	F	T	-
cholesterol	T	T	T	T	T	T	-
TNFα	T	T	T	F	F	F	--
triglycerides	F	T	T	T	T	T	-
hemoglobin	T	F	T	T	T	T	-
HDL-cholesterol	F	T	T	T	T	T	-
LDL-cholesterol	F	T	T	T	T	T	-
IGF-1	T	F	T	F	T	T	--

creatinine	T	T	T	T	T	T	-
monocytes	T	F	T	T	T	T	-
alkaline phosphatase	T	T	T	T	T	T	-
Hba1c	T	T	F	T	T	T	-
IL8	T	T	T	T	F	F	--
hematocrit / RBC	T	F	T	T	T	T	-
cystatin C	T	T	T	T	T	T	-
NT-proBNP	T	T	T	T	F	T	-
D-dimer	T	F	T	T	T	T	-
PAI1	T	T	F	F	F	T	--
urea	T	T	T	T	T	T	-
CD4/CD8 ratio	T	F	F	T	F	T	--
IL15	T	T	F	F	F	T	--
bilirubin	T	T	T	T	T	F	-
MCV	T	F	T	T	T	T	-
MCHC	T	F	T	T	T	T	-
C-peptide	F	F	T	T	T	T	--
IL1-β	T	F	T	F	F	T	--

table S1.2: Scoring system for e-score assignment (“research lab” biomarkers; non-epigenetic)

biomarker	1	2	3	4	5	6	e-score
telomere length (TLs):							
average TLs	T	T	T	F	F	F	--
telomere structure	T	T	F	F	F	F	---
shortest TLs	T	T	F	F	F	F	---
ROS	F	F	T	F	F	F	---
DNA damage	T	T	F	F	T	T	--
miRNA	T	F	F	F	F	F	---
TGF-β	T	T	T	F	F	T	--
mitochondrial dysfunction	F	F	F	F	F	F	---
autophagy (reduced)	F	F	T	F	F	T	---
EVs (extracellular vesicles)	F	T	F	F	F	F	---

telomerase activity	F	F	F	F	F	T	---
exRNA	F	F	F	F	F	F	---
gut microbiome	T	T	T	F	F	T	--
adiponectin	T	T	T	T	F	T	-
SIRT1	T	T	F	F	F	T	--
α-Klotho	T	T	F	T	F	T	--
GDF15	T	T	T	F	F	T	--
SIRT6	T	T	F	F	F	T	--
GDF11	T	T	T	F	F	T	--
CXCL1	T	T	F	F	F	T	--
skin microbiome	T	T	T	F	F	T	--

table S1.3: Scoring system for e-score assignment (“research lab” biomarkers; epigenetic)

biomarker	1	2	3	4	5	6	e-score
DNA methylation and aging clocks:							
Horvath clock	T	T	T	T	F	F	--
Hannum's clock	T	T	T	T	F	F	---
DNAm GrimAge	T	T	T	T	F	F	--
DNAm PhenoAge	T	T	T	T	F	F	--
Weidner clock	T	T	T	T	F	F	--
EpiTOC	F	F	T	T	F	F	---
Histone modifications:							
H4K20 methylation	F	F	F	F	F	F	---
H4K16 acetylation	F	F	F	F	F	F	---
H3K4 methylation	F	F	F	F	F	F	---
H3K9 methylation	F	F	F	F	F	F	---
H3K27 methylation	F	F	F	F	F	F	---
non-coding RNA expression profiles	T	F	F	F	F	F	---
chromatin remodeling	T	T	F	F	F	F	---

table S1.4: Scoring system for e-score assignment (none-blood physical capability and organ function)

biomarker	1	2	3	4	5	6	e-score

physical capability:							
grip strength	T	T	F	F	T	F	--
walking speed	T	T	F	F	T	F	--
standing balance	T	T	F	F	T	F	--
timed up and go test	T	T	F	F	T	F	--
organ function:							
body mass index	T	T	F	F	T	F	--
systolic blood pressure	T	T	F	F	T	F	--
atherosclerosis	T	T	F	F	T	F	--
cognitive function	F	T	F	F	T	F	---
muscle mass	T	T	F	F	T	F	--
bone health	T	T	F	F	T	F	--
lung function	T	T	F	F	T	F	--
waist circumference	T	T	F	F	T	F	--
general well being							
general decline	T	T	F	F	T	F	--

table S1.5: Scoring system for e-score assignment (senescence related biomarkers)

biomarker	1	2	3	4	5	6	e-score
SASP:							
IL-6	T	T	T	T	F	T	-
IL-7	T	T	F	F	F	T	--
IL-15	T	F	F	F	F	T	---
IL-8	T	F	T	F	F	F	---
CCL3	T	T	F	F	F	T	--
CCL4	T	T	F	F	F	T	--
GDF-15	T	T	T	F	F	T	--
activin a	T	T	F	F	F	T	--
cell ciycle arrest:							
p53	F	T	F	T	F	T	--
p21	F	T	F	T	F	T	--
p16	F	T	T	T	F	T	--

SA-βGal	F	F	F	F	T	F	---
SAHF:							
H3K9Me2	F	F	F	F	F	F	---
HP1y	F	F	F	F	T	T	---
lamin b1	F	F	F	F	T	T	---
cell morphology	F	F	F	F	T	T	---
